# Identification of key genes and multiple molecular pathways of metastatic process in prostate cancer

**DOI:** 10.7717/peerj.7899

**Published:** 2019-10-17

**Authors:** Lihuang Guo, Mingyue Lin, Zhenbo Cheng, Yi Chen, Yue Huang, Keqian Xu

**Affiliations:** 1 Department of Laboratory Medicine, The Third Xiangya Hospital, Central South University, Changsha, Hunan, People’s Republic of China; 2 Department of Laboratory Medicine, Xiangya School of Medicine, Central South University, Changsha, Hunan, People’s Republic of China

**Keywords:** Localized prostate caner, Metastaic prostate cancer, Metastasis, Differentially expressed genes

## Abstract

**Background:**

Cancer metastasis is well known as the most adverse outcome and the major cause of mortality in cancer patients, including prostate cancer (PCa). There are no credible predictors, to this day, that can reflect the metastatic ability of localized PCa. In the present study, we firstly identified the differentially expressed genes (DEGs) and molecular pathways involved in the metastaic process of PCa by comparing gene expressions of metastaic PCa with localized PCa directly, with the purpose of identifying potential markers or therapeutic targets.

**Methods:**

The gene expression profiles (GSE6919 and GSE32269) were downloaded from the Gene Expression Omnibus database, which contained 141 tissue samples, including 87 primary localized PCa samples and 54 metastaic PCa samples. After data processing, DEGs were identified by R language using the Student’s *t*-test adjusted via the Beniamini–Hochberg method. Subsequently, the gene ontology functional and pathway enrichment analyses of DEGs were performed and the protein–protein interaction network was constructed. Hub genes were identified using the plug-in cytoHubba in Cytoscape software by MCC and degree. Furthermore, validation and prognostic significance analysis of the hub genes were performed by UALCAN and gene expression profiling interactive analysis (GEPIA).

**Results:**

A total of 90 DEGs were identified between localized and metastaic PCa, which consisted of 47 upregulated and 43 downregulated genes. The enriched functions and pathways of the DEGs include catabolic process, cell cycle, response to steroid hormone, extracellular matrix (ECM)-receptor interaction and vascular smooth muscle contraction. A total of 10 genes were identified as hub genes and biological process analysis of hub genes showed that cell cycle phase, cell division, and mitotic cell cycle process were mainly enriched. The expression of hub genes were confirmed in metastaic PCa when compared with localized PCa tissues by The Cancer Genome Atlas database. Moreover, the disease-free survival analysis of hub genes revealed that these genes may play an important role in invasion, progression or recurrence. Therefore, these hub genes might be the key genes contributed to tumor progression or metastasis in PCa and provide candidate therapeutic targets for PCa.

**Conclusions:**

The present study identified some DEGs between localized and metastaic PCa tissue samples. These key genes might be potential therapeutic targets and biomarkers for the metastaic process of PCa.

## Introduction

Prostate cancer (PCa) is the most frequently diagnosed cancer and the fifth leading cause of cancer death in men, with 1.3 million new cases and 359,000 associated deaths worldwide in 2018 (Bray et al., 2018). The 5-year survival rate for metastaic PCa rapidly decreases to 30% despite the rate for localized PCa is almost 100% (Litwin & Tan, 2017). Therefore, cancer metastasis is the last thing that patients and doctors want to see. Usually, localized PCa spread to lymph nodes at first and then to the bones, liver or lungs (Wang et al., 2018). Mechanisms on how PCa develop and metastasize have been extensively investigated, which mainly include the process of PCa metastasis (Weidle et al., 2019), the biomolecules such as miRNAs and enzymes involved in metastasis (Pan et al., 2019; Wu et al., 2018) or aberrant glucose metabolism of metastaic PCa (Pichla et al., 2019). However, the mechanisms still remains incomplete for limitation by the failure to holistically reproduce each individual element of the metastatic cascade in PCa metastasis (Berish et al., 2018). Moreover, there is lack of easily accessible biomarkers which can accurately predict the metastatic potential of localized PCa. Therefore, it is necessary for us to figure out the differences about the biology between localized and metastatic PCa for developing new prognostic markers and therapeutic targets.

Microarray technology and bioinformatic analysis have been also widely applied, in order to explain the differences of gene expression which can help us better understand mechanisms about prostate tumorigenesis and progression (Huang et al., 2017). However, previous studies mainly focused on analysis of localized PCa and normal tissue samples. In the present study, we downloaded two gene expression profiles (GSE32269 and GSE6919) from the Gene Expression Omnibus database, which contain localized and metastaic PCa tissue samples. Then gene expression profiles were analyzed, and the differentially expressed genes (DEGs) were identified between localized PCa and metastaic PCa. Subsequently, gene ontology (GO) analysis, Kyoto Encyclopedia of Genes and Genomes (KEGG) pathway analysis and protein–protein interaction (PPI) analysis of DEGs were performed. Finally, a total of 90 DEGs and 10 hub genes were identified, which may be potential prognostic markers and therapeutic targets for PCa.

## Materials and Methods

### Microarray data

Two gene expression datasets (GSE32269 and GSE6919) were downloaded from GEO database (http://www.ncbi.nlm.nih.gov/geo) according to the following criteria: (a) the samples included metastaic PCa samples and primary PCa samples; (b) the file type of raw gene expression dataset is CEL; (c): the platform file contained probe ID, gene symbol and entrez gene ID. GSE32269 was based on GPL96 platform (Affymetrix Human Genome U133A Array) which contained 51 samples, including 22 primary localized PCa samples and 29 metastaic Pca samples. GSE6919 was based on GPL8300 platform (Affymetrix Human Genome U95 Version 2.0 Array) which contained 90 samples, including 65 primary localized PCa samples and 25 metastaic PCa samples.

### Data processing and DEGs screening

The raw CEL data were downloaded and standardized by the Affy package of R language (Gautier et al., 2004). According to the annotation files, each probe ID of expression matrix was replaced to corresponding gene symbol and if there were multiple probes that corresponded to the same gene, the average value was calculated using R language for further analysis. Then the genes of each dataset were screened using the limma R package and genes with a adjusted *P*-value < 0.05 and |log_2_fold change (FC)| > 1 were considered DEGs (Smyth, 2004). DEGs of two datasets were integrated using an online webtool, Venn diagrams (http://bioinformatics.psb.ugent.be/webtools/Venn/), and the integrated DEG lists of upregulated and downregulated were saved for subsequent analysis.

### GO and KEGG pathway analysis of DEGs

The DAVID6.8 database (https://david.ncifcrf.gov/) was used to perform GO functional and KEGG pathway analysis of the intergrated DEGs (Dennis et al., 2003). And the GO functional analysis of integrated DEGs involves three parts: biological process (BP), cell component and molecular function. *P* < 0.05 was considered to indicate a statistically significant difference.

### PPI network and module analysis

A protein–protein interaction network of the intergrated DEGs was constructed by STRING with a default medium confidence (0.4). Then, Cytoscape software (version 3.7.1) was used to visualize PPI networks (Shannon et al., 2003). Finally, the most significant module of PPI network was found by the plug-in molecular complex detection and the pathway analysis was performed in this module (Pichla et al., 2019). *P* < 0.05 was considered to indicate a statistically significant difference.

### Hub genes selection and analysis

The plug-in cytoHubba was used to rank nodes in a network by their network features through 12 topological analysis methods, including MCC, DMNC, MNC and EPC (Chin et al., 2014). The hub genes were selected as following standards: (a) top 30 nodes of the network by MCC analysis method; (b) the degree of gene is ≥10. Subsequently, the connection between hub genes and their co-expression genes was analyzed using the cBio Cancer Genomics Portal (cBioPortal), an open platform for exploring multidimensional cancer genomics data (Cerami et al., 2012), and then the BP of the hub genes was analyzed.

### Validation and prognostic significance analysis of the hub genes

The expressions of hub genes were compared among different nodal metastasis status of PCa using UALCAN, an interactive web portal to analyze The Cancer Genome Atlas (TCGA) gene expression data deeply (Chandrashekar et al., 2017). The PCa samples were divided into three groups (metastases in lymph nodes vs no regional lymph node metastasis vs normal). Then the prognostic significance of each hub gene was performed by gene expression profiling interactive analysis (GEPIA) (Tang et al., 2017). *P* < 0.05 was considered to indicate a statistically significant difference.

## Results

### Microarray data normalization and identification of integrated DEGs

**Figure 1:**
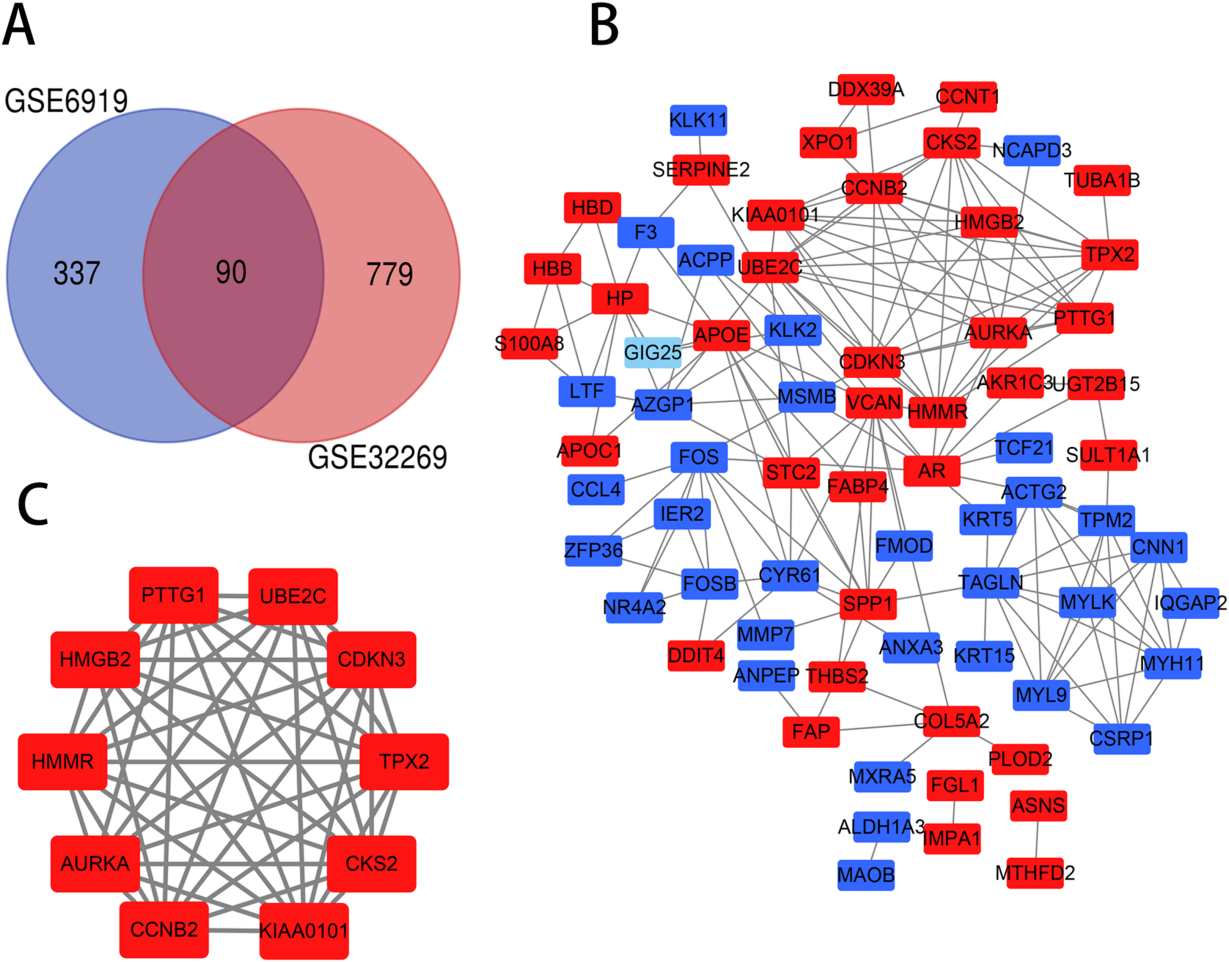
Venn diagram, PPI network and the most significant module of DEGs. (A) DEGs were selceted with a |log_2_fold change (FC)| > 1 and *P*-value < 0.05 between the GSE6919 and GSE32269. The two datasets showed an overlap of 90 genes. (B) The PPI network of DEGs was constructed using Cytoscape. (C) The most significant module was obtained from PPI network. Upregulated genes are marked in light red and downregulated genes are marked in light blue.

The PCa chip expression datasets GSE32269 and GSE6919 were normalized by R language, then the DEGs were screened using the limma R package (adjusted *P* < 0.05 and |log_2_fold change (FC)| > 1). The GSE32269 dataset contained 869 differential genes, including 706 upregulated genes and 163 downregulated genes. The GSE6919 dataset contained 427 differential genes, including 319 upregulated genes and 108 downregulated genes. Then an overlap of 90 DEGs was identified from the two profile data sets, which was displayed by Venn diagram (Fig. 1A), including 47 upregulated genes and 43 downregulated genes in the metastaic PCa compared to localized PCa (Table S1).

### GO and KEGG pathway analysis of DEGs

The top five GO terms of upregulated and downregulated DEGs were shown in Table 1. And we can find that the upregulated genes were mainly enriched in regulation of catabolic process, extracellular region part and monocarboxylic acid binding, while the downregulated genes were mainly enriched in response to steroid hormone, extracellular region part and cytoskeletal protein binding. As for the pathway analys, the upregulated DEGs were significantly enriched in extracellular matrix (ECM)-receptor interaction and oocyte meiosis pathway, while the downregulated DEGs were significantly enriched in vascular smooth muscle contraction pathway.

**Table 1:** Gene ontology and pathway enrichment analysis of the upregulated and downregulated genes.

Category	Term	Count	*P*-value
Downregulated			
BP	GO:0048545~response to steroid hormone	7	2.05E-04
BP	GO:0051384~response to glucocorticoid	5	3.07E-04
BP	GO:0009605~response to external stimulus	14	4.39E-04
BP	GO:0003008~system process	12	0.00422
BP	GO:0009719~response to endogenous stimulus	10	0.00691
CC	GO:0044421~extracellular region part	26	5.13E-07
CC	GO:0070062~extracellular exosome	22	9.15E-07
CC	GO:1903561~extracellular vesicle	22	9.97E-07
CC	GO:0031988~membrane-bounded vesicle	22	5.85E-05
CC	GO:0015629~actin cytoskeleton	6	0.00703
MF	GO:0008307~structural constituent of muscle	4	1.35E-04
MF	GO:0008092~cytoskeletal protein binding	9	7.22E-04
MF	GO:0005516~calmodulin binding	5	0.00104
MF	GO:0005200~structural constituent of cytoskeleton	4	0.00228
MF	GO:0003779~actin binding	6	0.00240
KEGG	hsa04270: vascular smooth muscle contraction	3	0.0367
KEGG	hsa00360: phenylalanine metabolism	2	0.0436
Upregulated			
BP	GO:0009894~regulation of catabolic process	9	4.85E-05
BP	GO:0051726~regulation of cell cycle	11	1.83E-04
BP	GO:0050790~regulation of catalytic activity	17	2.00E-04
BP	GO:0065009~regulation of molecular function	18	5.94E-04
BP	GO:0051246~regulation of protein metabolic process	16	0.00136
CC	GO:0005615~extracellular space	16	1.64E-05
CC	GO:0044421~extracellular region part	24	4.12E-04
CC	GO:0005829~cytosol	22	5.14E-04
CC	GO:0005576~extracellular region	24	0.00548
CC	GO:0072562~blood microparticle	6	1.09E-04
MF	GO:0033293~monocarboxylic acid binding	4	7.06E-04
MF	GO:0043168~anion binding	6	0.00107
MF	GO:0031406~carboxylic acid binding	5	0.00108
MF	GO:0005539~glycosaminoglycan binding	5	0.00259
MF	GO:0005102~receptor binding	10	0.0172
KEGG	hsa04512: ECM-receptor interaction	4	0.00509
KEGG	hsa04114: oocyte meiosis	4	0.00998

### PPI network and the most significant module analysis

The PPI network of DEGs was constructed (Fig. 1B) and the most significant module was obtained using Cytoscape (Fig. 1C). The functional analyses of genes involved in this module were analyzed using DAVID. Results showed that genes in this module were mainly enriched in mitotic cell cycle process, perinuclear region of cytoplasm, protein kinase binding and oocyte meiosis (Table 2).

**Table 2:** GO and KEGG pathway enrichment analysis of DEGs in the most significant module.

Category	Term	Count	*P*-value
BP	GO:1903047~mitotic cell cycle process	8	3.99E-08
BP	GO:0000278~mitotic cell cycle	8	7.14E-08
BP	GO:0007049~cell cycle	8	3.05E-06
BP	GO:0051301~cell division	6	5.42E-06
BP	GO:0000280~nuclear division	6	5.80E-06
CC	GO:0048471~perinuclear region of cytoplasm	4	0.00583
CC	GO:0005654~nucleoplasm	6	0.0222
CC	GO:0005829~cytosol	6	0.0374
MF	GO:0019901~protein kinase binding	3	0.0360
MF	GO:0019900~kinase binding	3	0.0443
KEGG	hsa04114: oocyte meiosis	3	0.00249

### Hub gene selection and analysis

**Figure 2:**
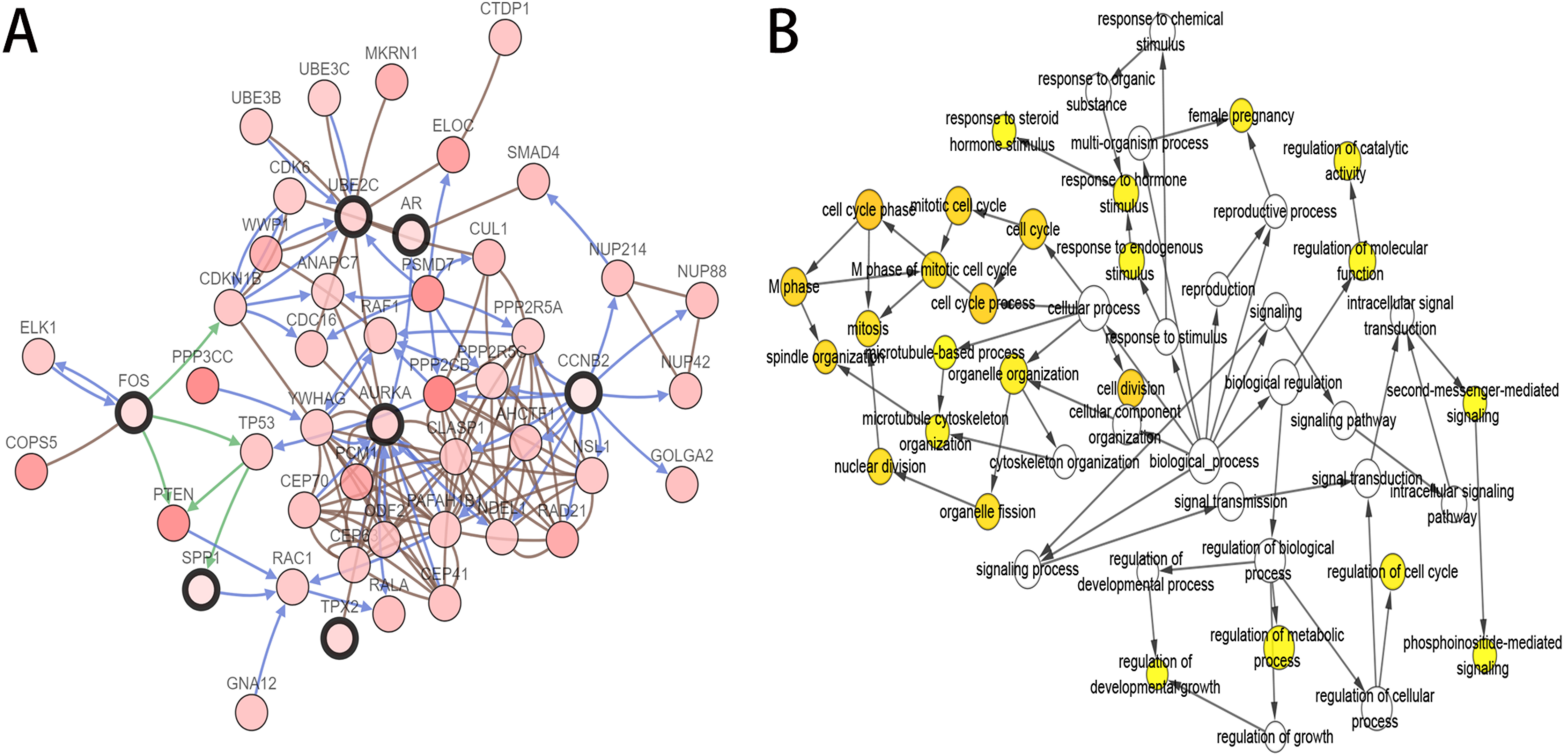
Interaction network and biological process analysis of the hub genes. (A) Hub genes and their co-expression genes were analyzed using cBioPortal. Nodes with bold black outline represent hub genes. Nodes with thin black outline represent the co-expression genes. (B) The biological process analysis of hub genes was constructed using BiNGO. The color depth of nodes refers to the corrected *P*-value of ontologies. *P* < 0.01 was considered statistically significant.

As the criteria described above, a total of 10 genes were identified as hub genes in the PPI network. Nine of these hub genes were upregulated including UBE2C, Cyclin B2 (CCNB2), CKS2, HMMR, androgen receptor (AR), CDKN3, TPX2, AURKA, SPP1 and only one gene, FOS, was downregulated. A network of the hub genes and their co-expression genes was constructed using cBioPortal online platform (Fig. 2A) and the BP analysis of the hub genes is shown in Fig. 2B.

### Validation and prognostic significance analysis of the hub genes

**Figure 3:**
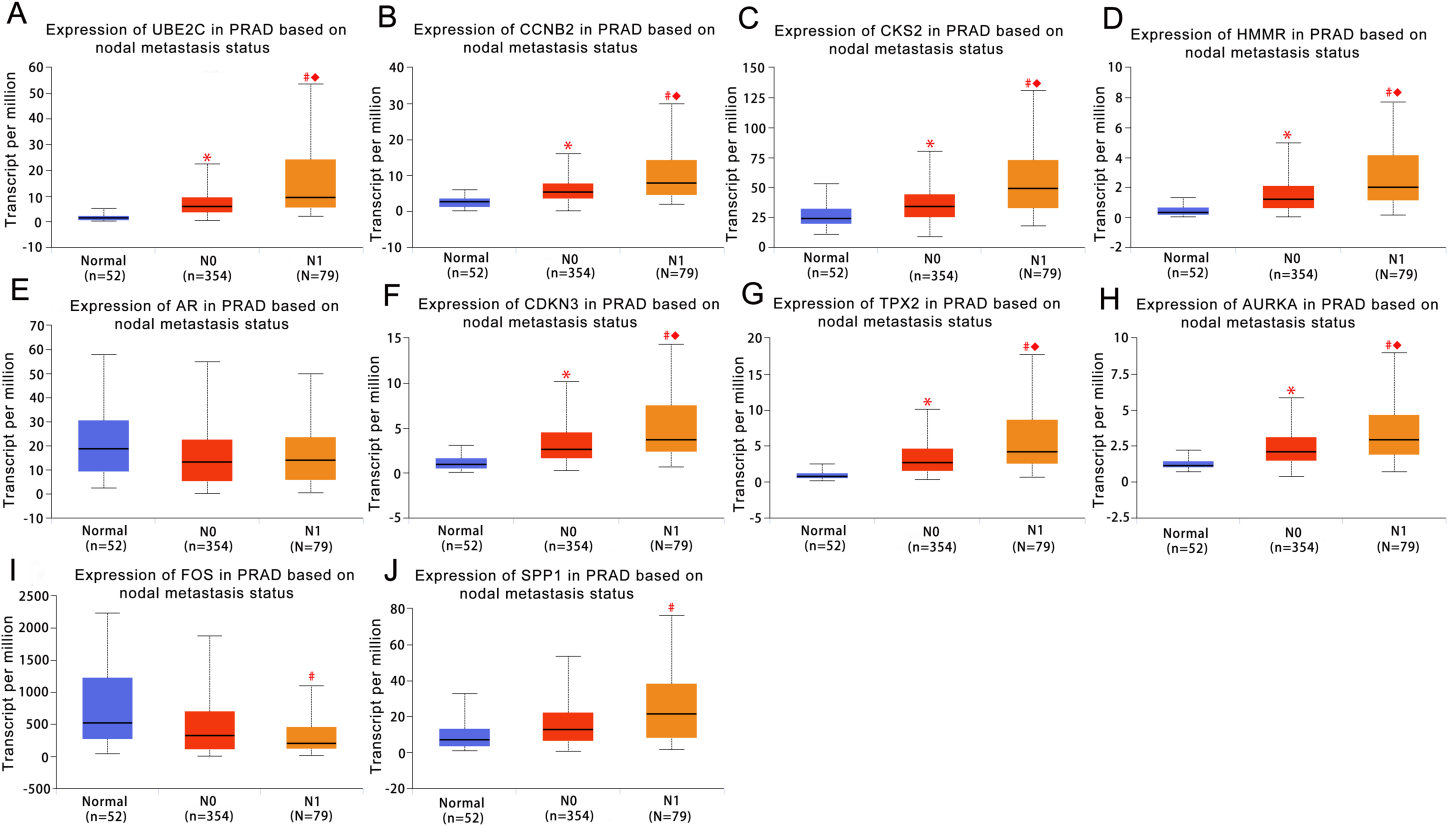
Box plots of gene expression values for hub genes in normal prostate samples, primary prostate cancer and metastatic prostate samples. (A) UBE2C (B) CCNB2 (C) CKS2 (D) HMMR (E) AR (F) CDKN3 (G) TPX2 (H) AURKA (I) FOS (J) SPP1. Normal, normal prostate samples; N0, no regional lymph node metastasis samples; N1, metastases in lymph nodes samples. *Normal vs N0 and *P* < 0.05; # Normal vs N1 and *P* < 0.05; ◆N0 vs N1 and *P* < 0.05. *P* < 0.05 was considered statistically significant.

UALCAN was used to analyze the hub gene transcript expression in the 476 samples which are derived from the TCGA project. The samples included 52 normal samples, 345 localized PCa samples and 79 metastaic PCa samples, so expression values in normal samples, localized and metastatic samples were compared. As shown in Fig. 3, the expression of the majority of hub genes in metastaic tumor samples were significantly elevated when compared with localized tumor samples or normal samples. These findings suggested our results of the identified candidate hub genes are reliable.

**Figure 4:**
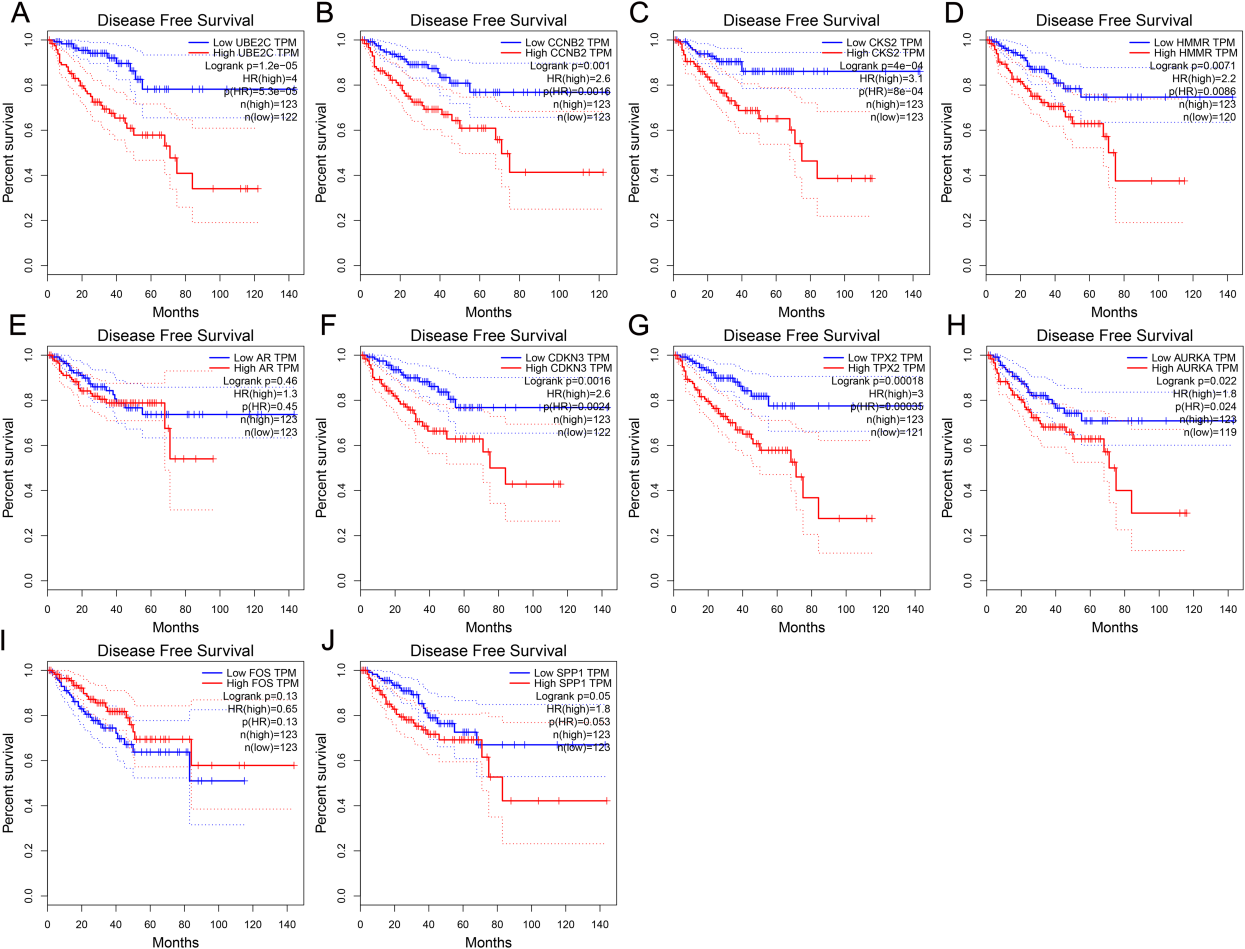
Disease-free survival curves of hub genes in prostate cancer from TCGA database. (A) UBE2C (B) CCNB2 (C) CKS2 (D) HMMR (E) AR (F) CDKN3 (G) TPX2 (H) AURKA (I) FOS (J) SPP1. *P* < 0.05 was considered statistically significant.

To further investigate whether these hub genes contributed to the prognostic in patients, GEPIA, an online tool with data sourced from TCGA and GTEx, was used to analyze the disease-free survival of these hub genes in PCa. As shown in Fig. 4, high expression of UBE2C, CKS2, CCNB2, HMMR, CDKN3, TPX2 and AURKA showed worse disease-free survival in PCa patients, while the disease-free survival of the other three genes were not significant, which revealed that UBE2C, CCNB2, CKS2, HMMR, CDKN3, TPX2 and AURKA were associated with prostate tumor progression and might be used as tumor progression predictors for prostate tumor patients.

## Discussion

Over the last decades, many researches including microarray bioinformatics were focused on the molecular mechanisms of PCa generation, development and metastasis, however, the biological programs underlying this complex process still remain unclearly. Different from former studies, we firstly adopted an integrated bioinformatics approach to directly compare the the differences in gene expression between localized PCa and metastaic PCa. An overlap of 90 DEGs was identified, including 43 downregulated genes and 47 upregulated genes. Then, GO enrichment and KEGG pathway analyses were performed to show us how alterations in DEGs expression affect the biological pathways involved in the development of PCa. The BP of upregulated genes were mainly enriched in regulation of catabolic process, regulation of cell cycle and regulation of protein metabolic process, while the downregulated genes were mainly enriched in response to steroid hormone and response to glucocor ticoid. Previous studies have reported that cancer cells possess different metabolic pathways when compared with healthy cells, in order to meet a fast growing need (Sikander et al., 2019). In addition, recent studies showed that metastasizing cancer cells require metabolic changes depend on the environment in which they reside (Fendt, 2019). Moreover, steroid hormones have far-ranging biological impacts involved in PCa initiation, development, metastasis and treatment (Agbo & Lambert, 2019; Snaterse et al., 2017). So, all these studies support our results. The most significant module of PPI network were mainly enriched in mitotic cell cycle, cell cycle and cell division, which also involved in tumorigenesis or progression (Sawada et al., 2019).

Hub genes, namely UBE2C, CDKN3, TPX2, CCNB2, AURKA, CKS2, HMMR, AR, SPP1 and FOS were identified in analyzing the PPI network of DEGs, indicating these genes may be vital in metastaic PCa. UBE2C belongs to ubiquitin-conjugating enzyme family for the destruction of mitotic cyclins and cell cycle progression. Dastsooz et al. (2019) reported that UBE2C is over expressed in all 27 cancers including PCa and significantly higher in late-stage tumors, and also participates in tumor progression or invasion by regulating the cell cycle, apoptosis and metastasis. UBE2C has also been found over expressed in androgen-independent PCa (Wang et al., 2009) and castration-resistant PCa (Chen et al., 2011). Similarly, CDKN3 (also called CDI1 or KAP) is a member of the dual specificity protein phosphatase family, and plays a critical role in PCa via regulating cell cycle and DNA replication signaling (Yu et al., 2017). CCNB2 is a member of the B-type cyclin family, which contains CCNB1 and CCNB2, serves a key role in progression of G2/M transition. Previous studies have shown that CCNB2 is highly expressed in a variety of tumors, such as colorectal adenocarcinoma, breast cancer and even serves as a poor prognostic biomarker in non-small-cell lung cancer (Park et al., 2007; Shubbar et al., 2013; Qian et al., 2015).

The androgen receptor gene is located on the X chromosome at location Xq11-12. It is well known that AR represents a vital driving force in the development and progression of prostate (Beretta & Zaffaroni, 2019) and the signaling pathway of AR plays important roles in prostate carcinogenesis and metastatic or androgen-independent progression of the disease (Tien & Sadar, 2019). In addition, Mills (2014) reported that AR involves in PCa development by affecting genomic stability and DNA repair. However, we found that the expression of AR in metastaic PCa was not significantly elevated compared with localized tumor samples (Fig. 3). In fact, there is no significant expression difference between and among metastaic PCa, localized PCa and normal tissue samples. Moreover, we assessed the expression of AR in relation to disease-free survival of PCa, and there is no significant difference too. The same results were found when we used another online cancer database tool (the data didn’t show). A possible reason for this is that AR expression can be heterogeneous in PCa patients (Koochekpour, 2010). It was proposed that AR has several genetic alteration forms involved in metastatic or progression of PCa including genomic amplification of AR, hypersensitive AR resulting from point mutations, promiscuous mutant AR protein activated by nonandrogenic ligands and AR-polymorphisms changing the response to androgen (Heinlein & Chang, 2004). Structural and functional AR abnormalities might lead to an alteration of the AR activity, which result some patients have high AR activity while the expression is normal, and further research is needed to confirm the exact molecular mechanisms.

Another interesting finding of hub genes was the expressions of FOS and SPP1. There is no significant difference between localized PCa and normal tissue samples, but when compared between the metastaic PCa and normal samples, the expression of FOS was decreased significantly and SPP1 was increase significantly, which indicating these genes might be potential predictors for the metastatic ability of PCa. SPP1, also known as osteopontin, an ECM proteinis involved in tumorigenesis and cancer progression and has been shown to be a key role of PCa metastasize to the bone (Desai, Rogers & Chellaiah, 2007). Pang et al. (2019) reported that SPP1 is significantly associated with survival in several forms of cancer, and plasma SPP1 levels may serve as markers for stage, grade and early tumor progression in PCa. FOS is one of the first studied proto-oncogenes and has been implicated as regulators of cell growth, differentiation and apoptosis (Piechaczyk & Blanchard, 1994). In the present study, FOS was observed to be downregulated, indicating its underlying tumor metastasis suppressor effect, which was consistent with Zhang et al. (2007) finding that FOS has a proapoptotic function by repressing the antiapoptotic molecule to induce apoptosis in PCa cells.

Differing from a previous study (Liu et al., 2018), our study included a larger sample size and presented a more comprehensive and systematic study. But there are still some certain limitations to the present study. PCa is extremely heterogeneous and multiple molecular subtypes of primary and metastatic PCa have been characterized (Harris et al., 2009; Cancer Genome Atlas Research Network, 2015). However, the subclass of PCa datasets in this study is not known, which may lead a number of important genes ignored and restrict the ability to draw a valid result. The absence of experiments verifying the expression of hub genes was also a limitation in the present study.

## Conclusions

In conclusion, our study identified some significant genes and biological pathways using an integrated bioinformatics analysis, which could improve our understanding of the metastaic process of PCa. These genes might be potential predictors and therapeutic targets for the metastaic PCa. However, further studies are required to validate these findings.

## Supplemental Information

10.7717/peerj.7899/supp-1 Supplemental Information 1 Integrated differentially expressed genes of prostate cancer.

## Additional Information and Declarations

### Data Availability

The following information was supplied regarding data availability: The raw data and code are available at: Guo, Lihuang (2019): Identification of key genes and multiple molecular pathways of metastatic process in prostate cancer. figshare. Dataset. DOI 10.6084/m9.figshare.9194273.v1.
