# Supplementary material for: Identification of key genes and multiple molecular pathways of metastatic process in prostate cancer
Source: PeerJ. 2019 Oct 17;7:e7899. doi: 10.7717/peerj.7899 (PMC6800981; doi:10.7717/peerj.7899)
Supplement: Supplemental Information 1 [file peerj-07-7899-s001.docx]

| **Integrated DEGs** | **Gene names** |
| --- | --- |
| Upregulated | *VCAN MTHFD2 TPX2 FGL1 DDX39A SULT1A1 HMMR COL5A2 FAP DDIT4 IMPA1 CLDND1 SLC39A14 FABP4 SERPINE2 SPP1 HBD UBE2C STC2 APOE CCNB2 AP2B1 AKR1C3 ACSM1 UGT2B15 XPO1 CCNT1 GRAMD4 DOCK4 KIAA0101 HP SERPINA3 HPRT1 GMFG S100A8 AR PLOD2 THBS2 CDKN3 ASNS PTTG1 HBB APOC1 HMGB2 AURKA CKS2 TUBA1B* |
| Downregulated | *TAGLN IER2 ALDH1A3 ACTG2 MOXD1 MMP7 CSRP1 ZFP36 TCF21 MYL9 F3*  *NCAPD3 PAGE4 NR4A2 ALOX15B KLK2 MAOB TPM2 PCP4 CNN1 ANXA3 FOS*  *MYH11 FOSB KRT5 NEFH MYLK MSMB CCL4 FMOD MXRA5 MEIS2 AZGP1*  *ACPP KLK11 SYNM LTF BCAS1 CYR61 ANPEP ADIRF KRT15 IQGAP2* |

### Integrated DEGs, Integrated differentially expressed genes
